# Monitoring Hypoxia Induced Changes in Cochlear Blood Flow and Hemoglobin Concentration Using a Combined Dual-Wavelength Laser Speckle Contrast Imaging and Doppler Optical Microangiography System

**DOI:** 10.1371/journal.pone.0052041

**Published:** 2012-12-18

**Authors:** Roberto Reif, Jia Qin, Lei Shi, Suzan Dziennis, Zhongwei Zhi, Alfred L. Nuttall, Ruikang K. Wang

**Affiliations:** 1 Department of Bioengineering, University of Washington, Seattle, Washington, United States of America; 2 Oregon Hearing Research Center, School of Medicine, Oregon Health and Science University, Portland, Oregon, United States of America; University of Navarra, Spain

## Abstract

A synchronized dual-wavelength laser speckle contrast imaging (DWLSCI) system and a Doppler optical microangiography (DOMAG) system was developed to determine several ischemic parameters in the cochlea due to a systemic hypoxic challenge. DWLSCI can obtain two-dimensional data, and was used to determine the relative changes in cochlear blood flow, and change in the concentrations of oxyhemoglobin (HbO), deoxyhemoglobin (Hb) and total hemoglobin (HbT) in mice. DOMAG can obtain three-dimensional data, and was used to determine the changes in cochlear blood flow with single vessel resolution. It was demonstrated that during a hypoxic challenge there was an increase in the concentrations of Hb, a decrease in the concentrations of HbO and cochlear blood flow, and a slight decrease in the concentration of HbT. Also, the rate of change in the concentrations of Hb and HbO was quantified during and after the hypoxic challenge. The ability to simultaneously measure these ischemic parameters with high spatio-temporal resolution will allow the detailed quantitative analysis of several hearing disorders, and will be useful for diagnosing and developing treatments.

## Introduction

Several hearing disorders such as noise-induced hearing loss, age related hearing loss, sudden sensorineural hearing loss, tinnitus and Ménière’s disease, have been related to cochlear ischemia [1]–[9]. Normal blood supply to the cochlea is important for establishing the endocochlear potential and sustaining production of endolymph. High altitude and hypoxia have been demonstrated to affect inner ear functions [10], [11]. Understanding the mechanisms underlying the pathophysiology of the cochlear microcirculation and oxygenation is of fundamental clinical importance, and may enable more effective understanding and management of these disorders [12].

Cochlear ischemia is an understudied area of research due to the lack of tools available. Previous methods have been *ex vivo*, invasive to the cochlea, require the use of contrast agents or have poor spatial or temporal resolutions. It has been difficult to obtain information regarding cochlea ischemia in humans given that the invasiveness of the techniques carries the risk of increasing the functional loss. Histological techniques can provide morphological information about the cochlea vasculature; however, these results are difficult to interpret [13]. The microsphere method is a single time point quantitative technique which requires a large number of samples for statistical purposes [14]–[16]. Laser Doppler flowmetry has been used to measure the relative changes of cochlear blood flow (CBF) during loud sound exposure [17], and during the use of vasodilators [18]. This technique has been limited because it averages the changes in blood flow within a large volume of the cochlea (a hemisphere with a ∼1–1.5 mm radius); therefore, there is uncertainty about which vessels contributed to the measured signal [19].

In this paper we propose to use a synchronized dual-wavelength laser speckle contrast imaging (DWLSCI) system [20] which is combined with a Doppler optical microangiography (DOMAG) system, to determine several cochlea ischemic parameters. DWLSCI is capable of determining the relative change in blood flow, and changes in the concentrations of oxyhemoglobin (HbO), deoxyhemoglobin (Hb) and total hemoglobin (HbT). DOMAG uses a spectral-domain optical coherence tomography (OCT) system, and it enables determining the absolute blood flow within single vessels. DOMAG has previously been used to image cochlear blood flow [21]–[23]. Both of these systems are non-invasive to the cochlea and do not require the use of contrast agents. In this study, the combined system was used to quantify the ischemic parameters in the cochlea of five mice subjected to a systemic hypoxic challenge.

## System and Methods

### 1.1. Ethics Statement

The experiments were performed on C57BL/6 adult mice of 20–30 g weight. All procedures were performed with approval from the Institutional Animal Care and Use Committee at the University of Washington (Protocol #4262-01).

### 1.2. System Setup

**Figure 1:**
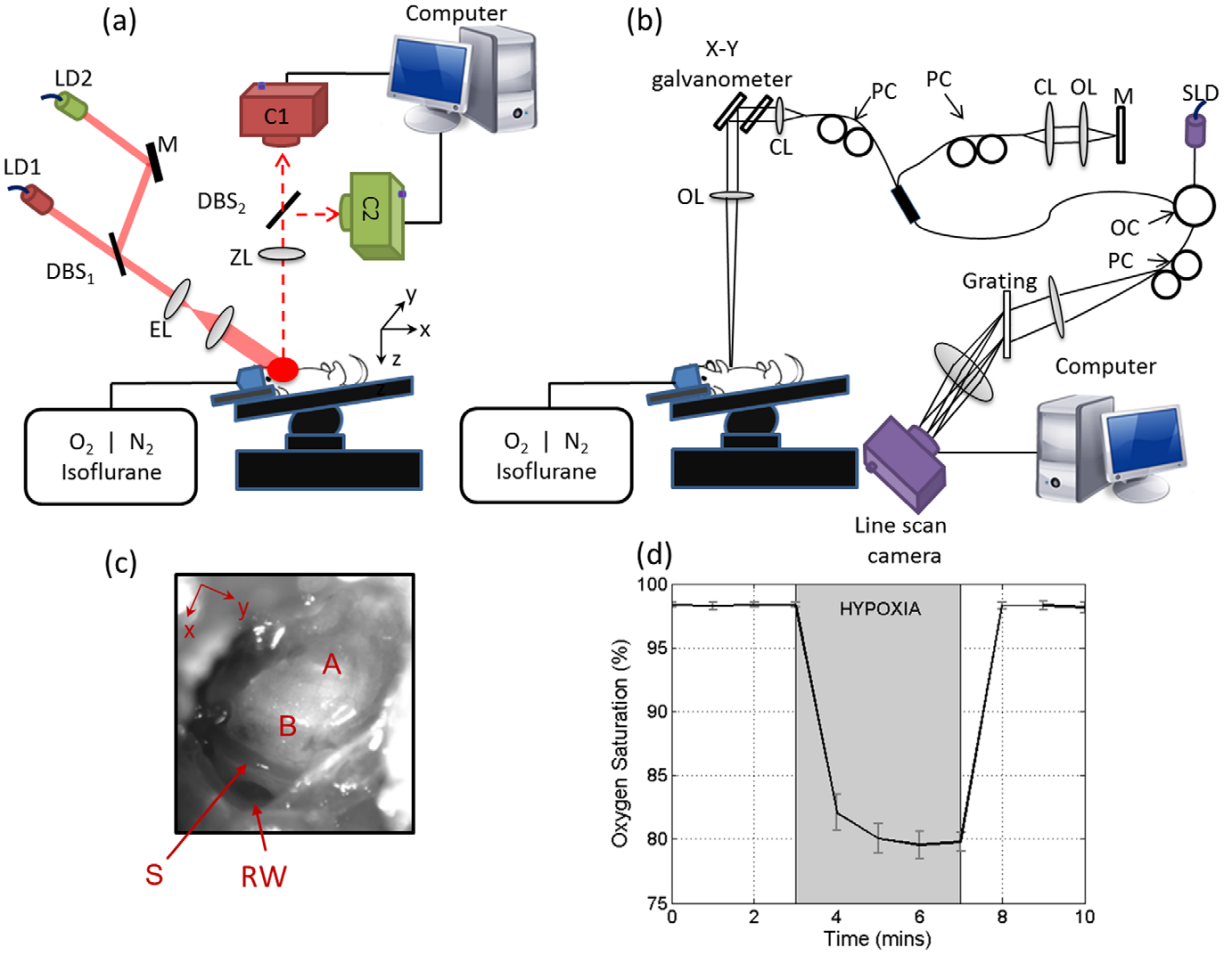
DWLSCI and DOMAG system setup. (a) Synchronized dual wavelength laser speckle contrast imaging system setup. C = camera, DBS = dichroic beam splitter, EL = expanding lens, LD = laser diodes, M = mirror, N_2_ = nitrogen, O_2_ = oxygen, ZL = zoom lens. (b) Doppler optical microangiography system setup. CL = collimator, OC = optical coupler, OL = objective lens, PC = polarization controller, SLD = superluminescent diode. (c) Ventral view image of the cochlea after opening the bulla. The regions of the apical (A) and basal (B) turns, the stapedial artery (S) and the round window (RW) can be observed. (d) Mean and standard deviation of the systemic oxygen saturation of the five mice, indicating the hypoxia between minutes 3 and 7.

Two systems were developed and combined. The DWLSCI and DOMAG are presented in Figures 1a and 1b, respectively. Although we present the systems in two separate figures (for ease of view), they are one integrated system. Therefore, when the animal subject is positioned, both systems are collecting data over the cochlea simultaneously without requiring the animal to be moved. A variation of the combined system, which contains a single camera laser speckle contrast imaging system, has previously been used to monitor a mouse burn injury [24].

#### 1.2.1. Dual-Wavelength Laser Speckle Contrast Imaging (DWLSCI)

The synchronized DWLSCI system setup is presented in Figure 1a. The system contains two collimated laser diodes with a wavelength of λ_1_ = 780 and λ_2_ = 825 nm. The lasers were combined coaxially by a dichroic beam splitter DBS_1_ (99% transmission and reflection for λ_1_ and λ_2_, respectively). Both lasers were expanded and uniformly illuminated over the sample at an incident angle of ∼60° [25] from the tissue normal direction. The backscattered light was transmitted to a zoom lens and to a dichroic beam splitter DBS_2_, with the same parameters as DBS_1_. The light from each wavelength was collected by a CMOS camera (Basler A504k). Both cameras were acquiring images at 50 frames per second using 1000×1000 pixels. The exposure time of the camera was set to 10 ms, which is optimized to achieve high contrast for speckle flow imaging [26].

#### 1.2.2. Doppler Optical Microangiography (DOMAG)

OCT is a technique capable of determining the microstructural composition of biological tissues. DOMAG is a method based on spectral domain OCT [27], which provides the detailed microvasculature and blood flow information. Figure 1b shows a schematic diagram of the DOMAG system, which is illuminated by a superluminescent diode light source (central wavelength 1310 nm, bandwidth 56 nm). The theoretical axial resolution in air is ∼13 µm. The light is divided into two paths using a 2×2 optical coupler. One of the light paths transmits light towards a mirror (reference arm), and the other light path transmits light towards the cochlea (sample arm). In the sample arm, the light is coupled into an optical system which contains a collimator, a pair of galvo scanners, and an objective lens with a 30 mm focal length, providing a lateral resolution of ∼10 µm. The light reflected from the sample and reference arm are recombined and transmitted to a home-built spectrometer. The spectral resolution was 0.141 nm which provides an imaging depth of ∼3.0 mm.

The scanning pattern and the methods used to process the data have previously been described [23], [28]. Briefly, the pair of galvo scanners scans the focused beam across the cochlea, with one scanner moving in the X-direction and another scanner moving in the Y-direction, as defined in Figure 1c. The camera had a line scan rate of ∼5 kHz, and each frame contained 2000 A-lines that span ∼1.5 mm.

Before the initiation of the experiment, a three-dimensional data set was acquired for each cochlea, which consisted of 240 discrete locations on the Y-direction, where five B-frames were collected at each location and averaged together (240 locations x 5 average = 1200 B-frames total). The acquisition rate was two frames per second. The three-dimensional data cube contained 1024×2000×240 (z-x-y) voxels which took 10 minutes to acquire. This data set was used to calculate the Doppler angle of each vessel as described below. During the experiment, 1200 B-frames were acquired in the same cross-section position for a period of 10 minutes.

### 1.3. Animal Preparation

Five mice were anesthetized with 1.5% isoflurane in oxygen enriched air (20% oxygen/80% medical air). The anesthesia was administered using a face mask, and the animal breathed freely throughout the experiment. The head of the mouse was immobilized onto an imaging platform to minimize motion artifacts. The right cochlea was exposed by ventral surgical dissection as previously described [21], [23], [29]. An incision was made through the midline of the neck, and the left submandibular gland and posterior belly of the digastric muscle were removed by cauterization. The jugular was also ligated. The positions of hypoglossal and facial nerves and the sternocleidomastoid muscles were used to identify the location of the bulla. For this study, the previous surgical procedure was slightly modified. To allow a large field of view for the laser speckle light to reach the cochlea at the 60° angle, and to enable the DOMAG system to image the basal turn of the cochlea, the auditory bulla opening was widened by resection of the ventral as part of the boney annulus of the tympanic membrane, which was not previously accomplished. A ventral view of the cochlea is presented in Figure 1c.

### 1.4. Experimental Protocol

After the surgery to expose the cochlea was completed, the mouse was switched from oxygen enriched air to 20% oxygen/80% nitrogen (normoxia) under 1.5% isoflurane prior to the start of the data acquisition. A pulse oximeter (Starr Life Sciences, Oakmont PA) was placed on the mouse thigh to measure the systemic oxygen saturation (SpO_2_). A normoxia baseline was maintained for 3 minutes, then the oxygen concentration was lowered approximately by half and the nitrogen was balanced accordingly using a gas-proportioning meter in order to achieve severe hypoxia (∼80%) as determined by the SpO_2_ measurements. The hypoxic stimulus was maintained for 4 minutes followed by a 3 minute recovery period in which the oxygen and nitrogen were switched back to the normoxic conditions (20% oxygen/80% nitrogen). The mean and standard deviation values of the SpO_2_ measured for all five animals are presented in Figure 1d. The shaded box indicates the time duration of the hypoxic stimulus. The ischemic parameters were measured in five animals using the combined DWLSCI and DOMAG system. Oxygen saturation measurements were recorded at one minute intervals, DWLSCI measurements were recorded every 20 ms (50 frames per second), and DOMAG frames (x-z cross-sectional images) were collected every 0.5 seconds (2 frames per second) throughout the 10 minute procedure.

Using the DOMAG system, we were able to obtain three-dimensional images of the cochlea with micrometer resolution [30]. The cochlea structural images allow for the identification of the different scalas as described by Dziennis et. al. [23]. These images were used to identify the location of the apical and basal turn in the cochlea, and were co-registered with the DWLSCI images.

### 1.5. Data Analysis

#### 1.5.1. Dual-Wavelength Laser Speckle Contrast Imaging (DWLSCI)

The images acquired with the 780 nm camera were used to extract the relative change in cochlear blood flow. The speckle contrast image was obtained by quantifying the blurring of the speckle pattern over the integration time of the camera. Briefly, the spatial speckle contrast maps (K) were calculated as the ratio of the standard deviation (σ_s_) to the mean intensity (<I>) for a 5×5 pixel binning window. The relationship between the speckle contrast and dynamic features of the speckle is expressed in Equation 1, where the relative change of blood flow is derived from the model reported in [31], [32]:
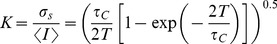
(1)where T is the exposure time of the camera (10 ms); and τ_c_ is the correlation time which is assumed to be inversely proportional to a measure of the velocity of the scattering particles. To reduce noise, every five flow velocity frames were averaged together.

Although it is theoretically possible to relate the correlation times to the absolute speed of the red blood cells; this has been challenging to do in practice given that the number of moving particles that the light interacts with and their orientations are unknown [33], [34]. However, relative spatial and temporal measures of speed can be easily obtained from the ratios of the correlation times. Several equations describe the correlation times as inversely proportional to the flow velocity (*τ_c_* = α/V); where V is the flow velocity, and α is a constant that depends on several variables that are unknown a priori, and is a topic that has been extensively discussed within the speckle contrast community [35]–[37]. However, the use of correlation time to provide the absolute value of the flow velocity is still questionable at the present time.

Images acquired with both cameras were used to obtain the changes in the concentrations of Hb, HbO and HbT. Since the concentration changes of HbO and Hb, which is related to the tissue absorption properties, would cause variations in the diffuse reflectance, the Beer-Lambert model was used to estimate the changes in hemoglobin concentration [24], [25], [38], [39], as presented in Equation 2:
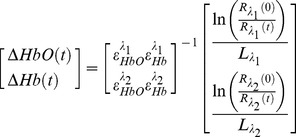
(2)where ε is the molar extinction coefficient of the absorber; R_λ1_(t) and R_λ2_(t) are the measured diffuse reflectance at the two wavelengths; R_λ1_(0) and R_λ2_(0) are the baseline diffuse reflectance values; L_ λ1_ and L_λ2_ are the path lengths of the light inside the medium and assumed to be constant, time-invariant and equal to 1.5 mm [38], [40]. The path length values are only used for the calculation of the changes in hemoglobin. The change in the total concentration of hemoglobin is obtained by ΔHbT = ΔHb+ΔHbO, and the value is assumed to be linearly proportional to the local blood volume [25]. To minimize noise on the reflectance images, every five reflectance frames were averaged together.

#### 1.5.2. Doppler Optical Microangiography (DOMAG)

The axial flow velocity can be derived from the phase difference (Δφ) between adjacent OCT A-lines. The phase difference is caused by the motion of blood cells inside blood vessels. The axial velocity can be obtained from:
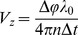
(3)where λ_0_ is the central wavelength of the light source (1310 nm), n is the refractive index of the tissue (1.35) and Δt is the time interval between adjacent A-lines (1/5000 Hz = 200 µs). The maximum axial velocity that can be measured is ±1.2 mm/s. The mean velocity of blood flow in spiral ligament vessels and stria vascularis has been previously reported as 0.12 mm/s and 0.08 mm/s, respectively [41]. To obtain the absolute velocity, it is important to determine the angle between the blood vessel direction vector and the vector of incident light, also known as Doppler angle. The angle is determined by measuring a three-dimensional data set of the sample [42], and is given by:
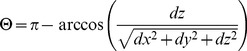
(4)where dx, dy and dz are the directional components of the blood vessels. The calculation of the blood flow velocity is obtained by:



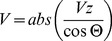
(5)Finally, the blood flow rate is obtained by the product of the velocity times the cross-sectional area of the blood vessel (A):
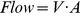
(6)


## Results

**Figure 2:**
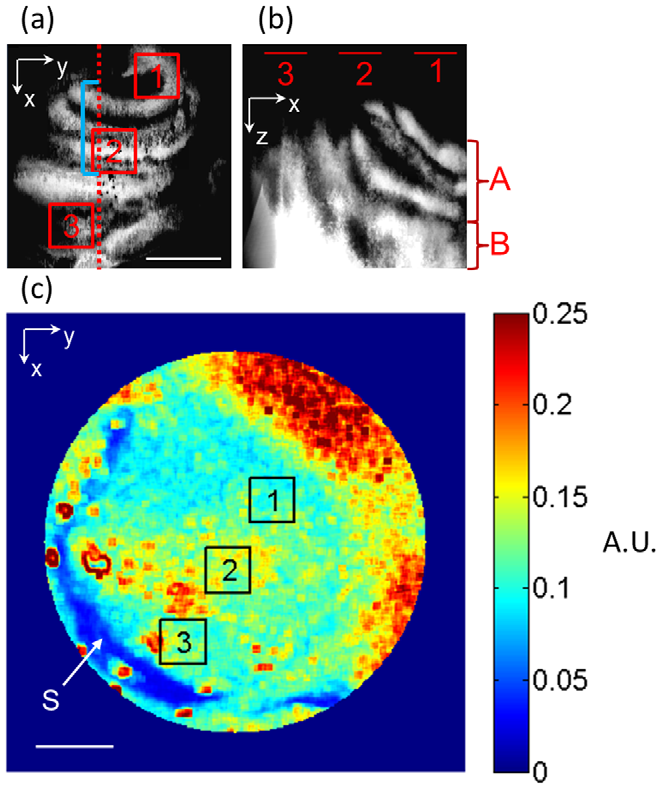
Localization of cochlear regions. (a) Maximum projection view of the scalas inside the cochlea in the z-direction (en-face). The red-dotted line is the cross-section visualized by the DOMAG systems during the hypoxic challenge. The blue line highlights the location of the lower apical turn. (b) Maximum projection view of the scalas inside the cochlea in the y-direction. The apical (A) and basal (B) turn regions have been indicated. (c) Laser speckle contrast image of the cochlea, highlighting the stapedial artery (S). Scale bar = 500 µm. Three regions of interest are shown. A.U. = arbitrary unit.

The DOMAG system was used to obtain both structural and microvascular images of the cochlea. The three-dimensional structure images enabled visualizing the scala vestibuli, tympani and media within the cochlea. These images were used to obtain a maximum projection view of the cochlea in the depth (*en face* or z) direction, as shown in Figure 2a which includes a depth of ∼1 mm. The cochlea in mice has 1.75 turns which contain the apical and basal turns [43]. We have selected three regions of interest (ROI) which include: 1) ROI1 at the apical turn close to the helicotrema, 2) ROI2 approximately at the lower apical turn and 3) ROI3 at the basal turn. It is important to mention that although ROI1 is located over the apical turn, part of the basal turn is located underneath it. This can be visualized in Figure 2b which presents a maximum projection view of the cochlea in the side (or y) direction for ∼0.5 mm, where the scalas belonging to the apical and basal turns have been indicated.

Figure 2c shows the speckle contrast image obtained using the DWLSCI system. The region outside the circle was removed because it was out-of focus and/or didn’t contain the cochlea. The stapedial artery is easily visualized and has high velocity flow as indicated by its low contrast image value. The ROI’s obtained from the OCT images were co-registered on the speckle contrast image.

**Figure 3:**
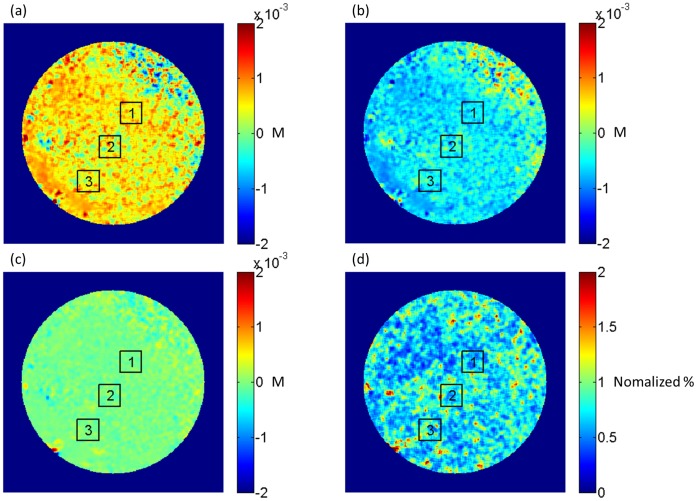
Image of changes in the ischemic parameters. Spatial map indicating the changes in the concentration of (a) deoxyhemoglobin, (b) oxyhemoglobin and (c) total hemoglobin at the last minute of the hypoxia. (d) Spatial map indicating the relative change in blood flow in normalized percent. Three regions of interest are shown. M = molar.

The spatial maps indicating the changes in the concentrations of deoxyhemoglobin, oxyhemoglobin, and total hemoglobin, and the relative change in blood flow at minute seven (last moment of the hypoxic challenge) of the experiment from one mouse obtained with the DWLSCI system are presented in Figure 3a, b, c and d, respectively. The relative change in blood flow was obtained by dividing the velocity map at a given time point by the velocity map at time point 0; therefore, a value of 1 indicates no change. It can be noted that throughout the cochlea there was an increase in Hb, a decrease of HbO and blood flow, and almost no change in HbT, which is indicated by the red, blue and green colormap, respectively. It is noticeable that the changes are fairly homogeneous throughout the cochlea.

**Figure 4:**
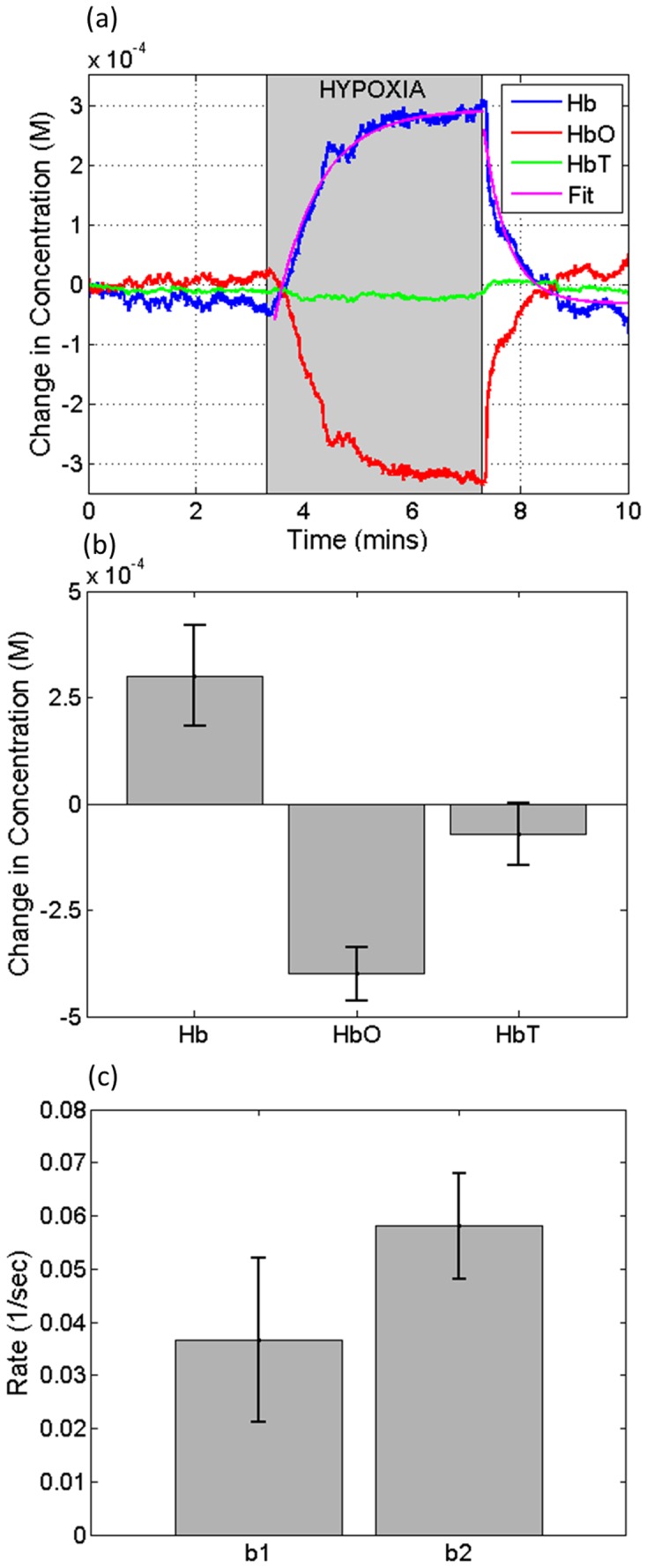
Changes in the concentration of Hb, HbO and HbT. (a) Change in the concentration of Hb, HbO and HbT through time. The purple curves are the fits of Eqn. 7 and 8 to the change in the concentration of Hb. The shaded area indicates the time points for the hypoxic stimulus. (b) Mean and standard deviation of the change in the concentrations of Hb, HbO and HbT at the last time point of the hypoxia phase. (c) Mean and standard deviation of the rate b1 and b2 from the change in concentration of Hb. M = molar.

Figure 4a presents the changes in the concentration of hemoglobin through time from a mouse in ROI 1. It can be observed that there is an increase and decrease in the concentrations of Hb and HbO, respectively, while HbT presents a slight decrease. Both Hb and HbO were fitted to an exponential model, given by:
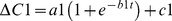
(7)

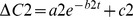
(8)


Equation 7 was used to fit the change in concentration (ΔC1) within the hypoxic stimulus (minutes 3 through 7), and Equation 8 was used to fit the change in concentration (ΔC2) after the hypoxic stimulus (minutes 7 through 10). The fitting parameters of these equations were a1, a2, b1, b2, c1, and c2. The value t indicates time in seconds. Figure 4a presents the curve fit ΔC1 and ΔC2 for the changes in the concentration of Hb (purple curve).

Figure 4b shows the mean and standard deviation of the last time point of the hypoxic stimulus (minute 7). These results indicate the animal to animal variability. However, for all mice, there was an increase in Hb, a decrease in HbO and a slight decrease in HbT.

Figure 4c shows the mean and standard deviation of the rate of concentration change b1 and b2 of Hb, obtained from Equations 7 and 8, respectively. Similar values were obtained for HbO (data not shown).

The results obtained from ROI2 and ROI3 (data not shown) were compared to ROI1. For all cases, the results were very similar and not statistically significant, indicating that (1) the changes are similar for all regions of the cochlea, and/or (2) the DWLSCI system has limitations where the volume of tissue averaged is large; therefore, providing similar results throughout the cochlea.

**Figure 5:**
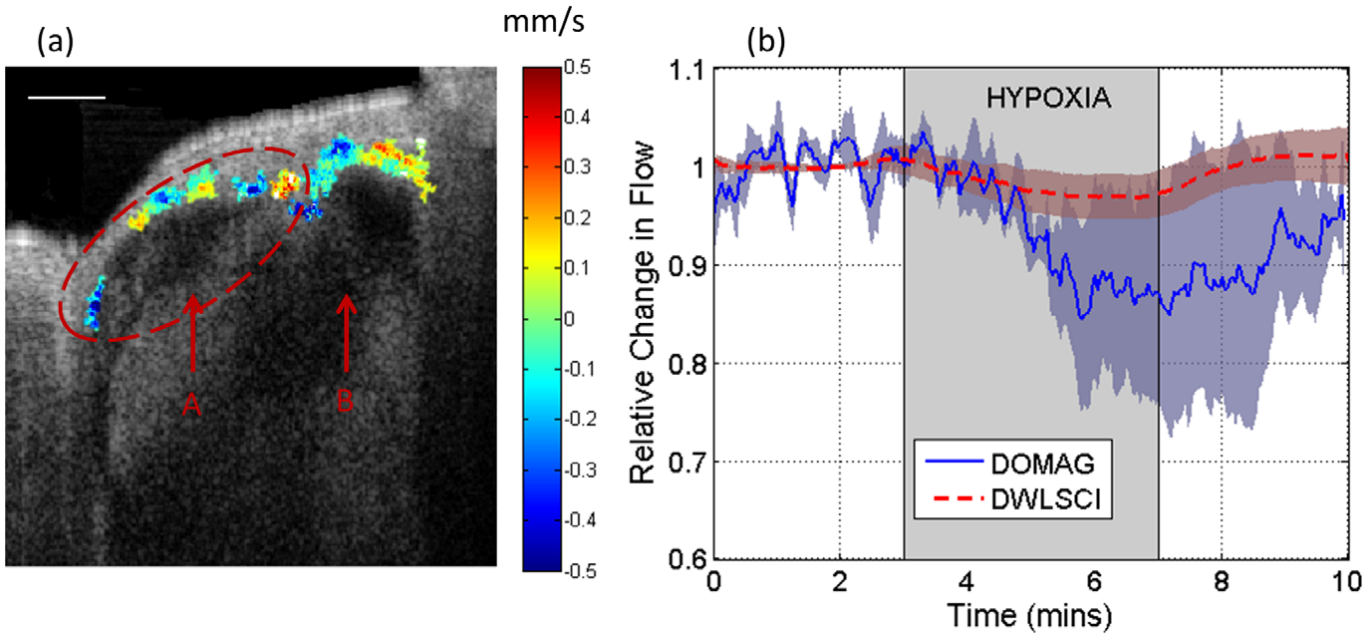
Changes in cochlear blood flow. (a) Structural image of the cross-section of the cochlea. The scalas of the lower apical (A) and basal (B) turns are indicated. The velocity obtained by the Doppler signal from the cochlea vessels is shown, and the colormap indicates the total velocity. Scale bar = 250 µm. (b) Relative change in cochlear blood flow measured by DWLSCI in ROI 2, and DOMAG indicated by the red oval in (a) and also highlighted by the blue line in Figure 2(a). The shaded area indicates the time points for the hypoxic stimulus.

The relative changes in blood flow rate were quantified for both systems. Figure 5a shows a cross-section structural image of the cochlea obtained from a location such as red dotted line in Figure 2(a). We can observe the scalas for the lower apical and basal turns. The total red blood cell velocity image was overlaid, to indicate the rate, location and direction of flow of the blood vessels. The blood flow rate was quantified for all the vessels in the lower apical turn. These vessels are highlighted inside the red dashed oval in Figure 5(a) as well as by the region shown in Figure 2(a) with a blue line. The mean and standard deviation relative change in flow for all mice is shown in Figure 5(b). The blue line indicates the flow acquired by the DOMAG system, and the red line indicates the flow acquired in ROI2 by the DWLSCI system.

## Discussion

During a hypoxic challenge, the SpO_2_ was reduced from >95% to ∼80%. Inside the cochlea, we observe that from a baseline measurement, there is an increase and decrease of Hb and HbO, respectively. The decrease of HbO is ∼20% larger than the increase of Hb, causing a slight decrease of HbT, as shown in Figure 4b. These values return close to their baseline values after the hypoxic challenge is over, as presented in Figure 4a. In general, the changes in the concentrations of Hb and HbO are inversely correlated, although the magnitude change of HbO is higher than Hb.

During and after the challenge, the changes in the concentrations of Hb and HbO follow an exponential trend, and have been fitted to a simple model given by Equations 7 and 8. These equations allow for the quantification of the rate of change in hemoglobin concentration during (b1) and after (b2) the hypoxic challenge. This model demonstrates that quantitative parameters may be extracted, and we expect that these could be used to assess the health of cochlea tissue. Further studies will analyze the rate change for different cochlea pathologies. Although the results presented in Figure 4c are from Hb, similar values were obtained for HbO (data not shown).

Cochlear blood flow was measured using both systems. In both cases the relative change in cochlear blood flow had a decrease due to the hypoxic challenge; however, the DOMAG results have a larger decrease than the DWLSCI system. An explanation for this discrepancy is that the region where the data is evaluated is not exactly the same for both systems (as shown in Figure 2(a)), where DOMAG calculates the flow across a cross-section in the surface of the cochlea, and DWLSCI collects the flow within an area (ROI2) which averages the signal through depth. Also, both systems have different spatial resolutions and penetration depths, and they have two and three dimensions for the DWLSCI and DOMAG, respectively. Further studies should be completed to properly compare the blood flow measurements from both systems.

It is also noted that the mean and standard deviation data of the DWLSCI system is smoother and smaller compared to its DOMAG counterpart. This is expected given that 1) the DWLSCI system averages the signal through a large volume of tissue, while the DOMAG data is high-resolution and is localized; 2) DOMAG can be sensitive to the pulsatile nature of the blood flow, which is not apparent in the DWLSCI signal; and 3) the selection of the DOMAG cross-section and the vessel segmentation is arbitrary based on the expert’s best estimate, which is another possible source of error. Although the trends in both systems are similar, their results are not directly comparable.

The blood flow results from the DWLSCI, were similar for all ROI’s. Previously it was demonstrated using DOMAG that the vessels in the apical turn close to the helicotrema (approximately ROI1) had an increase in cochlear blood flow [23]. However, the results presented in this study using DWLSCI indicate that there is a decrease in cochlear blood flow close to the helicotrema. We attribute the inconsistency to the difference in system resolution and penetration depths. DOMAG is a technique that obtains three-dimensional images with micrometer resolution, and enables obtaining the cochlear blood flow from individual vessels close to the surface. However, DWLSCI is a two-dimensional technique which averages the contributions from several vessels within a volume of tissue. Figure 2b presents a side view projection OCT image of the cochlea. We can observe that in ROI1, the apical turn is close to the surface, but the basal turn is underneath it. Therefore, the reflectance signal obtained by the DWLSCI system is influenced by both the apical and basal turn vessels on ROI1, and in average they may present a slight decrease in cochlear blood flow. Therefore, DWLSCI is not easily capable of separating the signal from the different turns in the depth dimensions, and may be a limitation of the system for cochlea studies.

A solution for separating the signal from the apical and basal turns, using the DWLSCI, would be to rotate the angle of incidence of the laser light and camera with the location of the cochlea. However, this presents a surgical challenge since there is a significant amount of tissue surrounding the cochlea, which would block the laser light. Another approach would be to develop a DWLSCI system which contains a thin fiber or an endoscope that could be used to better image the cochlea with different angles of incidence, or use mirrors that carefully control the incident angle of the light.

Equation 2 has limitations given that we assume the path lengths of the light inside the medium to be constant, time-invariant and equal to 1.5 mm. The cochlea is a non-homogeneous tissue, with highly scattering regions in the bone and low or non-scattering regions within the scalas. Therefore, it is very likely that the path length value used at different pixels with the reflectance image should be different, and that the path length also depends on the changes in the optical properties of the tissue through time. Further studies are required to properly characterize the influence of the path length in the changes in the concentration of hemoglobin for cochlea studies.

### Conclusion

A combined DWLSCI and DOMAG system is used to monitor several ischemic parameters in mouse cochlea due to a hypoxic challenge. The changes in the concentrations of Hb, HbO and HbT, as well as the relative changes in cochlear blood flow are monitored through time. During hypoxia there is an increase and decrease in Hb and HbO, respectively, and a slight decrease in HbT. The rate of change in the concentrations of Hb and HbO were quantified during and after the hypoxic challenge. The cochlear blood flow decreases for both DOMAG and DWLSCI systems; however, the magnitude of change is not exactly the same. We attribute this discrepancy to the differences in the regions analyzed and the differences in the resolutions and penetration depths of the systems. This combined system can be used to monitor the ischemic parameters in the cochlea and may enable understanding and treating several hearing disorders, such as noise-induced hearing loss, tinnitus and Ménière’s disease.
